# Unveiling the Challenges and Solutions: A Scoping Review of Maternal Healthcare Access in Rural Georgia

**DOI:** 10.7759/cureus.79238

**Published:** 2025-02-18

**Authors:** Pranitha S Kaza

**Affiliations:** 1 College of Sciences, Georgia Institute of Technology, Atlanta, USA; 2 Department of Policy and Research, Healthy Mothers, Healthy Babies Coalition of Georgia, Atlanta, USA

**Keywords:** indicators of maternal mortality, maternal health care, maternal health factors, maternal health policy, rural healthcare access

## Abstract

This literature review explores the influence of legislative policies on maternal healthcare accessibility in rural Georgia, where systemic barriers have created significant challenges for maternal health outcomes. Key legislation includes House Bill (H.B.) 1114 (2020), which expanded Medicaid postpartum coverage from 60 days to six months. S.B. 106, the "Healthy Babies Act," prioritizes telehealth and remote maternal health services, while H.B. 1037 (2023) proposes establishing the Georgia Commission on Maternal and Infant Health. Despite these advances, rural areas continue to face shortages of obstetric providers and facilities, financial barriers to care, and limited awareness of postpartum health risks. Recommendations include enhancing incentives for providers to accept Medicaid through faster reimbursement and expanding obstetric training programs to alleviate provider shortages. Extending Medicaid coverage to include chronic illnesses, mental health, and oral health up to one year postpartum is essential for comprehensive care. Additionally, fostering strong patient-provider relationships is necessary to strengthen postpartum health awareness and ensure open communication about potential health complications. These recommendations provide a framework for reducing disparities and improving maternal health outcomes in rural Georgia while offering insights into addressing broader rural healthcare challenges.

## Introduction and background

This literature review examines the current state of maternal health services in rural Georgia with a focus on the potential impact of proposed legislative policies and initiatives on improving accessibility. Comprehensively analyzing existing research will provide a thorough understanding of the challenges, opportunities, and potential improvements within the realm of maternal healthcare in rural Georgia. Maternal mortality, defined as the death of a woman during pregnancy, childbirth, or within 42 days of delivery due to pregnancy-related causes, is significantly higher in rural areas compared to urban counterparts [1]. Policies that address rural areas in the same manner as urban areas will not effectively resolve area-specific barriers to accessing maternal health services, such as transportation, healthcare provider shortages, and lack of infrastructure. This emphasizes the importance of location-specific prevention strategies to address disparities in rural regions [1]. While there has been significant growth in the establishment of maternal mortality and morbidity review committees (MMRCs) across the US, rural populations have been largely underrepresented in these efforts. Despite the higher health risks and limited access to healthcare faced by rural residents, only a small number of states have included rural representation in MMRCs. This lack of focus on rural communities may hinder the development of interventions specific to rural communities and thus emphasize the need for imminent policy intervention [2]. The adoption of a pregnancy status checkbox on US death certificates showed rural areas experiencing the largest increase in reported maternal deaths between 1999 and 2017. The analysis showed that rural areas saw a considerable increase, whereas large urban areas saw a decline [3]. The following sections will address the key factors that contribute to creating these barriers to accessing maternal health services and approaches that have been proposed to expand accessibility.

The World Health Organization defines the term "maternal health" as the health of the pregnant person during "pregnancy, childbirth, and the postnatal period" [4]. The absence of vital services that fulfill maternal health needs can deprive the pregnant person of safe, holistic prenatal, childbirth, and postpartum experiences [5,6]. The direct and indirect consequences of limited access to maternal health services include maternal injury to the physical and/or mental well-being and death; 60% of Georgia's pregnancy-related deaths are preventable, but continued negative health outcomes emphasize the inaccessibility of maternal health resources and services [7]. Rural women face higher risks of maternal and infant mortality compared to their urban counterparts, and more than half of rural US counties lack hospital obstetric services due to financial burdens leading to hospital closures [8,9]. In the US, the loss of hospital obstetric services in rural counties, particularly those not adjacent to urban areas, was associated with increases in out-of-hospital births, preterm births, and births in hospitals without obstetric units [10,11]. This highlights the need for targeted planning and policies to address gaps, specifically in rural obstetric care. A study that examined the closure of labor and delivery units (LDUs) in rural Georgia between the years 2012 and 2016 concluded that the closures disproportionately affected Black and low-income women more significantly, exacerbating the state's already high maternal mortality rates (MMRs). The study revealed that closures were linked to factors such as lower birth volumes, financial distress, and shortages of obstetric providers and emphasized the need for increased funding for maternity healthcare, financing LDUs, and addressing the workforce shortages in rural areas [12,13].

Additionally, children who are born in rural areas may have increased chances of experiencing exposure to health risks compared to their urban counterparts. One such study showed that low birth volume is a risk factor for maternal postpartum hemorrhage in rural hospitals [14]. Another study concluded that multiple factors, such as material circumstances, healthcare access, substance-use disorder, and pre-pregnancy health, influence maternal health and severe maternal morbidity (SMM) in rural Appalachian areas and identified the need for targeted interventions to address these factors and reduce SMM in rural communities [15]. Investigating the causes of poor health outcomes in the rural areas of Georgia provides insights into which maternal health services are available or lacking in rural areas. Policies in this realm include introducing Medicaid compensation for doulas, simplifying the process of becoming a licensed doula to encourage more individuals to pursue this role, deliberately training more "racially and culturally diverse obstetricians, nurse midwives, and doulas," and incentivizing "targeted investments in Black health care providers, students, researchers, educators, and communities" [16]. Examining how these interventions can parallel legislative policies and initiatives that are already in place will facilitate safe, rural maternal healthcare accessibility and services.

## Review

Methods

In conducting this literature review, a preliminary literature search was conducted with a focus on sources published between 2010 and 2024. Additionally, the literature search focused specifically on maternal healthcare in Georgia. Of the sources that were included upon the completion of the initial screening and full-text review stages, all sources were published during or after 2011 and used both quantitative and qualitative methods of research. Following the completion of the literature search and extractions, it became evident that there is a limited amount of literature available on the topic of rural maternal healthcare specific to Georgia.

Studies were included if they focused on maternal healthcare access and outcomes in rural US populations, with priority given to Georgia-based research, examined the impact of legislative policies, healthcare infrastructure, and financial barriers on maternal health, and/or included either quantitative or qualitative data on maternal health disparities, provider availability, or patient outcomes. Studies were excluded if they focused solely on urban maternal health without addressing rural disparities, were published before 2010, or were opinion-based articles without peer-reviewed evidence. Data were extracted from selected studies and categorized into key themes, including Medicaid coverage and financial barriers, provider shortages, telehealth accessibility, and infrastructure limitations. Some sources used for this review may span studies that were conducted on rural maternal populations in other parts of the US as well as other countries. Barriers and proposed policy interventions that have been identified in at least half or more of the sources reviewed have been articulated in the discussion section. The leading search term was any title with "maternal health" in relation to rural services or rural access. While the primary focus is on Georgia, comparisons to other rural regions and international contexts were included to provide broader insights into rural maternal healthcare challenges and potential solutions. This comparative approach helps contextualize Georgia's situation within larger trends and best practices.

Discussion

Georgia's Current Policies

Georgia's House Bill (H.B.) 1114, signed into law by Governor Brian Kemp in 2020, extends Medicaid coverage for low-income mothers from two months to six months postpartum. This law is aimed at improving maternal health outcomes, especially in a state with one of the highest MMRs in the US, which disproportionately affects Black women [17]. The extended coverage is intended to address gaps in care during the postpartum period, ensuring that mothers have access to necessary healthcare services, including mental health support, contraception, and chronic disease management. This policy is especially crucial in Georgia, where rural areas face unique challenges in accessing healthcare services, including Medicaid provider shortages and transportation barriers; however, challenges, such as limited access to Medicaid providers, low reimbursement rates, and the broader impact of Medicaid expansion decisions, continue to affect maternal healthcare outcomes. Similar barriers are observed in states like Alabama and Mississippi, where Medicaid access and financial constraints still significantly impact low-income mothers of the rural South [13,14]. The bill also includes provisions to expand Medicaid coverage for lactation services for new mothers and their children. The legislation is part of a broader effort to improve healthcare access for vulnerable populations in Georgia, addressing the healthcare gaps that contribute to poor maternal health outcomes. It represents a step forward in ensuring that new mothers have continued access to necessary healthcare services, which is expected to positively impact the long-term health and well-being of both mothers and their children [17].

Georgia Senate Bill 106 (S.B. 106), also known as the "Healthy Babies Act," was signed into law in May 2023. This legislation establishes a pilot program aimed at improving maternal and child health outcomes, particularly focused on those with Medicaid. The program, which began in Fiscal Year 2024, is intended to provide remote patient monitoring services. This initiative is designed to encourage expectant mothers to engage in prenatal care, potentially reducing the risk of preterm labor and improving birth outcomes. The bill was constructed in collaboration with the Georgia Department of Community Health and was a priority for the Senate Majority Caucus during the 2023 legislative session. The impact of this program is yet to be studied and monitored [18].

Georgia H.B. 1037, which was passed by the Georgia House of Representatives in February 2024, aims to establish the Georgia Commission on Maternal and Infant Health. This commission would be responsible for addressing maternal and infant health disparities in the state, including examining and providing recommendations for improving maternal health outcomes, with a particular focus on the intersection of health conditions, such as mental health issues, substance use, and physical complications, that affect maternal health. The bill has moved through the legislative process and is currently under review in the Senate. If passed, it would provide both a framework and funding to confront Georgia's maternal and infant health challenges [19].

Maternal Mortality in Rural Georgia

The US Census Bureau defines urban "as a continuously built-up area with a population of 50,000 or more" and rural "as what is not urban - that is, after defining individual urban areas, rural is what is left" [20,21]. The MMR is the number of maternal deaths for every 100,000 live births [22]. Not having enough clinical care centers and understaffing are significant barriers because "The reasons for these closures are multifactorial, and include workforce shortages, financial viability, low volume of patients, concerns over maintaining the knowledge base and skill sets of the obstetrical health care team required to provide high quality and safe care" [23]. Georgia having a significantly higher MMR puts pregnant persons giving birth in Georgia at a greater risk of death during birthing or within one year after giving birth [22]. Georgia's high MMR is influenced by several interconnected factors, including limited healthcare access, pronounced racial disparities, and socioeconomic challenges. Rural areas face significant shortages of maternal healthcare providers, forcing many women to travel long distances for prenatal and postpartum care. This geographic barrier is compounded by racial disparities, as Black women in Georgia experience MMRs that are more than twice as high as their White counterparts, potentially driven by systemic inequities in healthcare quality, implicit bias, and social determinants of health [22,24]. Socioeconomic factors also play a role; many low-income women face challenges such as inadequate health insurance, limited access to reliable transportation, and difficulty obtaining consistent medical care. These combined factors create a healthcare environment where timely and comprehensive maternal care is often out of reach, contributing to Georgia's persistently high MMR [22,23]. Often, Georgia is referred to as a "Maternity Care Desert" due to the absence of adequate care facilities and caregivers. In rural Georgia, maternal care facilities are sparsely located, and in those areas where they are present, "a full range of maternal health clinicians" are not available [7]. This usually leads to caregivers providing services that they are not experienced with and are not fully equipped to, which emphasizes the concerns surrounding under-qualified staffing in Georgia's rural maternal healthcare system [24].

Georgia Medicaid programs state that pregnant persons are eligible for a minimum of four antenatal visits that are fully covered. Despite this legislative initiative, only about 25% of pregnant persons in rural Georgia receive at least four antenatal visits other than labor and delivery in a clinical setting or hospital [6]. The reported reasons behind the low attendance for maternal health visits are physical barriers such as distance and limited public transportation, as well as inadequate options when scheduling visits around the patients' work hours. Only 15% of the rural maternal population received at least one of the four antenatal visits during the first trimester between 2007 and 2015 [6]. This statistic is immensely alarming as most prenatal conditions can be diagnosed as early as the first trimester or first 12 weeks of pregnancy. Delayed diagnosis of obstetrical conditions is one of the leading causes of maternal mortality in Georgia. Additionally, longer travel distances to maternity service sites with cesarean capabilities may negatively influence maternal and newborn health outcomes [25].

The results of a 2016 survey of maternal health services showed that only 23% of birthgivers in Georgia utilized postnatal care services in 2011 [26]. The Georgia Postpartum Coverage Report outlines that to compensate for chronic health conditions, such as hypertension and diabetes, and complications during delivery, birthgivers will have Medicaid coverage for up to one year postpartum [27]. The report mentions that this initiative will also encourage healthy pregnancy spacing and safe contraception, but there is no specific reference to postpartum mental health awareness. Postpartum health refers to both the physical and mental well-being of the birthgiver. Due to the limited maternal mental health services that are available to birthgivers, the lack of awareness about the importance of postpartum mental health poses another barrier to healthy maternal outcomes [26]. Another study found that while 75% of rural women gave birth at local hospitals, those with preterm births, clinical complications, or without access to intensive neonatal care at local hospitals were more likely to deliver at nonlocal hospitals, suggesting the potential physical barriers in accessing specialized care in rural settings [27].

Pregnant individuals in rural areas also experience a significantly higher risk of ICU admission and maternal mortality compared to those in urban areas, as identified in a study conducted across the US. Rural MMRs were nearly double those of urban areas. Despite an overall increase in maternal mortality in both settings, these findings emphasize the need for interventions that address the geographic disparities in maternal health outcomes [28,29]. Efforts should focus on improving resources and healthcare access in Georgia's rural areas to reduce maternal morbidity and mortality.

Barriers to Rural Maternal Health Services

There are several barriers that affect mothers in rural communities, some of which include insufficient or lack of maternal healthcare financing, inadequate communication between health professionals and pregnant persons, gaps in quality clinical care, staffing, infrastructural inaccessibility, and the reduced affordability of healthcare [22]. In rural Georgia, facilities that offer maternal health services are sparsely located, making distance and limited public transportation additional concerning factors; 35% of Georgia's counties lack obstetric care providers, and rural hospital closures have exacerbated this issue [30]. Some pregnant individuals travel 50 miles or more to reach the nearest maternity care facility, with some regions having even greater travel burdens [24]. A 2016 study of Georgia's maternal care system reported that "as of 2011, more than half (52%) of all (Primary Care Service Areas) outside the Atlanta (Atlanta Metropolitan Statistical Area) had an overburdening or complete absence of obstetric providers. These shortage areas lacked not only obstetricians but also delivering family medicine providers (89%) and delivering certified nurse midwives (70%)" [30]. Addressing the issues that create these boundaries requires an understanding of their underlying causes to ensure that new legislative actions are effective and that existing policies are properly amended. Louisiana is leading efforts by enacting laws that support maternal mental health, an example being the Perinatal Mood and Anxiety Disorders Act. This law aims to improve screening, treatment, and prevention of postpartum depression, a common and preventable complication; however, despite national recommendations for mental health screening, practices remain insufficient across the southeastern region of the US, highlighting the need for further policy development to improve access to mental health services for pregnant and postpartum women [31].

Health Literacy Among Pregnant Persons

Another significant barrier to positive health outcomes in rural maternal healthcare is the limited health literacy of pregnant persons. Multiple studies published within the past five years emphasize the inconsistency in communication between patients and obstetricians/gynecologists, which may lead to negative health outcomes [32]. Health literacy can be seen from a multifaceted approach because "Satisfaction with maternal care services is also strongly influenced and shaped by socio-demographic characteristics of women (the level of education, age, marital status, and economic status), a number of personal factors (values, attitudes, the threshold of pain, health literacy, and personal support), as well as perceived control and expectations formed on the basis of previous experiences and outcomes of previous pregnancies and births" [33]. Low health literacy is associated with delayed prenatal care initiation, increased rates of preventable pregnancy complications, and lower adherence to postpartum care recommendations. Many expectant mothers may struggle to understand critical health information, such as recognizing warning signs of preeclampsia or managing gestational diabetes, which means that they are less likely to engage in preventive care, leading to higher rates of emergency interventions during childbirth [32,33]. There are many different components to the patient-provider interaction that influence the pregnant person's understanding and interpretation of health, and healthcare providers may need to cater to the needs of pregnant people differently based on their distinct backgrounds, different experiences, and contrasting levels of pre-existing knowledge about maternal healthcare. A positive relationship is associated with a higher likelihood of patients feeling comfortable approaching their providers about health concerns or potential risk factors, fostering better communication and proactive care [33]. 

A 2024 study utilized a maternal mortality ratio to quantify the differences in outcomes based on education. Significant disparities were identified in maternal health outcomes between subgroups, specifically for rural women (maternal mortality ratio of 292) compared to urban women (maternal mortality ratio of 100) and for women with low education (maternal mortality ratio of 536) compared to those with high education (maternal mortality ratio of 85). This emphasizes the importance of addressing both healthcare access and social determinants, such as birthgivers' education and awareness pertaining to maternal health, to reduce maternal mortality and improve health outcomes in rural regions [34].

Alleviating Disparities in the Quality of Rural Maternal Health Services

A major component of all legislative policies put in place to assist pregnant persons in Georgia is Medicaid coverage. Even if more providers and locations for maternal health services are available, pregnant persons may still continue to be apprehensive about utilizing care due to the possibility of heightened medical costs because "financial incentives are effective for short-term but, alone, may not translate into long-term retention" [35]. Medicaid health insurance most often does not fully cover payments for pregnant persons with pre-existing health conditions or if chronic health conditions develop during pregnancy. A 2021 qualitative study also examined the birthgivers' views regarding maternal mental health services. The study explains that pregnant persons often consider these services a luxury rather than a necessity to one's well-being. The possibility of costs increasing over time, even with the presence of Medicaid coverage, halts many birthgivers from reaching out to maternal health service providers as a large portion of rural Georgia's population is under financial pressure [36]. Based on the data collected, the study concluded by making legislative recommendations that aim to alleviate the boundaries to accessible maternal health services [36]. 

Even though family physicians (FPs) do not primarily provide maternal health services, they are trusted individuals to many rural families. Expanding maternal healthcare education to FPs can increase the physical accessibility of maternal health services in rural areas [37]. The extent of FP involvement varies significantly from state to state, as most hospitals lack standardized procedural requirements for granting maternity care privileges to FPs. Recommendations include maintaining and improving maternal health education in family medicine residency programs, as they should continue training residents in maternity care and cesarean delivery to address the needs of rural communities [38]. This implementation also serves to relieve gaps in healthcare professionals' experiences and skill sets pertaining to maternal healthcare and practice [16]. This line of communication between patient and provider can be enhanced by certified nurse-midwives and certified midwives. Indeed, one such study found that for low-risk women planning midwife-led births, there is no significant difference in maternal or neonatal outcomes between rural and nonrural populations. This suggests that midwifery care in rural areas provides outcomes comparable to those in nonrural areas for this population [39]. Expanding the midwifery workforce is a recommended strategy to address not only workforce shortages but also gaps in patient education [40].

Addressing the Physical and Financial Barriers to Maternal Healthcare Access

Most Georgia physicians who accept Medicaid coverage are located around the urban and metropolitan areas. Providers in rural maternal communities may present with high out-of-pocket costs without Medicaid coverage and thus reduce financial accessibility for the population of pregnant persons in these areas [22]. For example, despite the implementation of the free maternal health policy under the National Health Insurance Scheme (NHIS), women in Ghana still faced out-of-pocket payments for certain services, highlighting the universal need for policy adjustments that are inclusive of chronic conditions and related health services to reduce if not eliminate high out-of-pocket costs [41]. A legislative initiative to expand Medicaid coverage to more maternal health providers across the state of Georgia would aid in reducing the financial apprehension around utilizing maternal health services [42]. Furthermore, to bridge the gap in health literacy between healthcare professionals and pregnant persons, it is vital to understand what resources are already available and to assess their effectiveness [32,43]. A 2014 study notes that even if pamphlets or flyers are available to a population, there does not exist a guarantee that the patient, caregiver, and/or family member will proactively access and make use of the resources that are available. The average health literacy rate being as low as 25% makes understanding health information difficult for many individuals [32]. Existing data assert the conclusion that information needs to be presented through a concise yet direct method. For example, the provider may have a direct conversation with the pregnant person about what to expect, what decisions they may need to make now or in the future, and how those decisions will translate into medical care, as conversation can facilitate a space for follow-up questions and discussion.

Incorporating proactive communication into the prenatal check-up routines ensures that pregnant persons living in rural areas are aware of which maternal health services are available, the significance of this care, and how to access the services [23]. A 2022 study discusses these points while emphasizing the necessity of the patient-provider relationship. With the presence of more direct communication between both parties, a higher level of trust is developed, increasing the likelihood that pregnant persons will feel comfortable asking questions, raising objections, and returning for the next appointment/visit [23]. California's introduction of the California Maternal Quality Care Collaborative (CMQCC) is a partnership between healthcare providers, public health professionals, and organizations involved in improving care for those who are pregnant or postpartum to provide support at varying levels, depending on the needs of the region of the state. The initiative also established a Maternal Data Center that allows hospitals to record and track clinical data in real time. Implementing a similar program across the US, including in the state of Georgia, is highly recommended as it will also provide the opportunity to tailor the program to the needs of rural counties [44].

Remote Maternal Health Services

Telehealth is an effective option for addressing rural-urban disparities in maternal care by improving accessibility, reducing the need for long travel distances, and providing specialized services that may not be available in rural areas. It enhances continuity of care through virtual visits, allowing for timely monitoring and interventions during critical maternal health periods [45]. While telehealth offers many benefits, it also comes with challenges, particularly in rural areas where limited broadband access and lack of digital literacy can hinder its effectiveness. Additionally, telehealth cannot fully replicate in-person care, as physical exams and certain diagnostic procedures require face-to-face visits. Issues such as inconsistent insurance reimbursement, technology barriers, and provider readiness further complicate its implementation. These limitations highlight the importance of investing in infrastructure, patient education, and policy reforms to maximize telehealth's potential in addressing maternal care disparities [46].

Integrated Maternal Healthcare

A 2005 study examining health system reforms in Georgia highlighted recommendations for improving maternal health that closely align with those of more recent studies, indicating that progress in this area has been limited over time [23,47,48]. The study emphasized the need for government-led interventions to address financial barriers and to strengthen primary healthcare infrastructure to integrate maternal healthcare with other healthcare disciplines, especially in rural areas. The alignment of these policy recommendations over nearly two decades suggests that while initial reforms, such as the introduction of the Rural Health Program, demonstrated early positive effects, sustainable and significant advancements in maternal health remain elusive, largely due to challenges such as inadequate funding, resource misallocation, and structural inefficiencies within the healthcare system [47,49,50]. Ideally, a multidisciplinary team of healthcare professionals works with the mother to establish comprehensive care when high-risk comorbidities are involved. This would allow for providing continuous support during preconception, pregnancy, and postpartum, though this would require extensive resource allocation in rural areas [51].

Future Directions

By addressing these multifaceted challenges, Georgia can move closer to a more equitable and effective maternal healthcare system for its rural communities. While this review provides valuable insights into the challenges and policies related to rural maternal healthcare based on secondary sources, incorporating local data, such as surveys or questionnaires, could offer more direct evidence of the specific barriers and needs in rural Georgia. Future research that includes primary data could further strengthen these findings and provide a more nuanced understanding of the region's unique challenges, as well as of the direct impact of policies on maternal health outcomes pre- and post-legislation.

## Conclusions

Maternal healthcare accessibility remains a significant issue in rural Georgia, with legislative action and organizational interventions consistently highlighted as key strategies to address the state's maternal health crisis; however, current interventions have not fully succeeded in improving the availability and diversity of services in rural areas, largely due to the persistent challenge of reducing medical costs across the full spectrum of maternal healthcare, encompassing pregnancy, childbirth, and postpartum care while driven by bureaucratic barriers. Strengthening incentives for healthcare providers to qualify for Medicaid, such as reducing reimbursement wait-times, expanding training options in obstetric specialty care, and introducing midwife coverage, remain key recommendations for attracting and retaining maternal health clinicians in rural areas. Improving access to rural Medicaid providers would help alleviate financial burdens, making essential services more affordable and accessible. Expanding postpartum coverage to address chronic illnesses, such as hypertension and diabetes, would ensure that mothers continue to receive the care they need beyond childbirth. Additionally, enhancing the patient-provider relationship by increasing trust and communication through consistent, culturally competent care is a consistent and essential strategy for improving patient outcomes, ensuring that the birthgiver feels supported, heard, and engaged in their own healthcare decisions throughout the maternal journey. The literature highlights the need for increased investment in rural hospitals to mitigate closures and enhance service availability, as well as transportation subsidies to alleviate one of the major barriers rural patients face in accessing maternal healthcare. Educating patients on the signs of postpartum complications and providing community-based support for maternal mental health will facilitate in preventing maternal mortality and improving overall health outcomes for pregnant persons.
